# Stage‐by‐stage exploration of normal embryonic development in the Arabian killifish, *Aphanius dispar*

**DOI:** 10.1002/dvdy.738

**Published:** 2024-09-19

**Authors:** Amena Alsakran, Rashid Minhas, Atyaf S. Hamied, Rod W. Wilson, Mark Ramsdale, Tetsuhiro Kudoh

**Affiliations:** ^1^ Department of Biosciences University of Exeter Exeter UK; ^2^ MRC Centre of Medical Mycology University of Exeter Exeter UK

**Keywords:** in vivo model, staging, temperature

## Abstract

**Background:**

Arabian killifish, *Aphanius dispar*, lives in marine coastal areas of the Middle East, as well as in streams that experience a wide range of salinities and temperatures. It has been used as a mosquito control agent and for studying the toxicities of environmental pollutants. *A. dispar'*s eggshell (chorion) and embryos are highly transparent and are suitable for high resolution microscopic observations, offering excellent visibility of live tissues.

**Results:**

In this study, the staging of normal embryonic development of *A. dispar* was described and investigated at different temperatures. Embryonic development was then examined under different thermal environments from 26 to 34°C. Our data suggest that temperature has a significant effect on embryonic development, with accelerated development at higher temperatures.

**Conclusion:**

*A. dispar* exhibits broad thermal tolerance and extended independent feeding capabilities, making it a promising model organism for toxicology and pathogenesis studies conducted over an extended period of time (12 days post‐fertilization).

## Key Findings

- Arabian killifish (A. dispar) has emerged as a promising model organism for biomedical research. Their transparent embryos enable precise, non‐invasive monitoring of developmental processes, making them an invaluable tool for studying genetics, immunological, drug efficacy and toxicity, as well as the impact of environmental pollutants on early life stages.
- Wide Temperature Tolerance: Killifish exhibit exceptional adaptability, thriving in a temperature range of 26°C to 34°C.
- Prolonged Independent Feeding Time: Killifish embryos demonstrate a significantly longer period of independent feeding (11 days post‐fertilization) compared to zebrafish (5 days post‐fertilization).

## 1 INTRODUCTION

The Arabian killifish (*Aphanius dispar*) is a small teleost fish that is distributed in coastal areas of the Eastern Mediterranean (Saudi Arabia, Iraq, Iran, the United Arab Emirates, Kuwait, Bahrain, Israel, Oman, and Yemen), the East African countries (Egypt, Djibouti, Somalia, Ethiopia, Eritrea, and Sudan), and Western India and south of Pakistan.^1^, ^2^, ^3^ This omnivore species tends to live in schools.^4^ The species has been used as an effective biological control agent for mosquito larvae.^5^ *A. dispar* can also live at extreme temperatures (37–40°C).^6^, ^7^ *A. dispar* is tolerant to a wide range of pesticides/toxins and can tolerate low levels of O_2_; therefore, it has been used as a biological model to evaluate eco‐toxicity.^7^, ^8^ Hence, *A. dispar* might be a suitable model to examine the effects of a wide range of environmental stressors on aquatic species or pathogens at a wide range of temperatures.

Small teleost fish embryos such as zebrafish and medaka have been used as models for studying genetic diseases,^9^, ^10^ infection,^11^, ^12^ ecotoxicology,^13^, ^14^ and drug testing^15^, ^16^, ^17^ as well as for assessing mechanisms of normal embryonic development and cell function.^18^, ^19^, ^20^ Daily these fish lay many eggs in the lab throughout the year, with the eggs rapidly developing body and organs within a few days. The development process can be easily observed due to the small size and the transparency of the embryo.^21^ Though zebrafish and medaka have been very powerful models for various studies, it is still necessary to use other fish models for different purposes. For instance, zebrafish embryos reach the independent feeding time at 5 days post‐fertilization, and the chorion of medaka has hair fibers. Moreover, zebrafish cannot survive above 34°C^22^; therefore, they are not ideal for higher temperature studies or drug testing for a longer period of time. It is also desirable to develop a fish model that can be used for investigating environmental changes and pollution in estuarine, marine, and high‐salinity environments. Here we report that the *A. dispar* embryo can become a valuable model for studying embryonic events with a range of different temperatures 12 days. In addition, the *A. dispar* embryo and chorion are highly transparent, offering an attractive model system for both high resolution and high throughput imaging purposes. This study investigated the step‐by‐step development of *A. dispar* embryos, delineating key developmental milestones from the maternal to hatching stages.

## 2 RESULTS

### 2.1 Normal embryonic development of *Aphanius dispar*

The following descriptions and dissecting stereomicroscope photographs of *A. dispar* provide an illustration of the stages for the formation of discernible embryonic structures during development. The *A. dispar* embryos were raised at 28°C for normal embryonic development.

#### 2.1.1 Stage 0.5 (0.5 hpf): early 1‐cell stage

**FIGURE 1:**
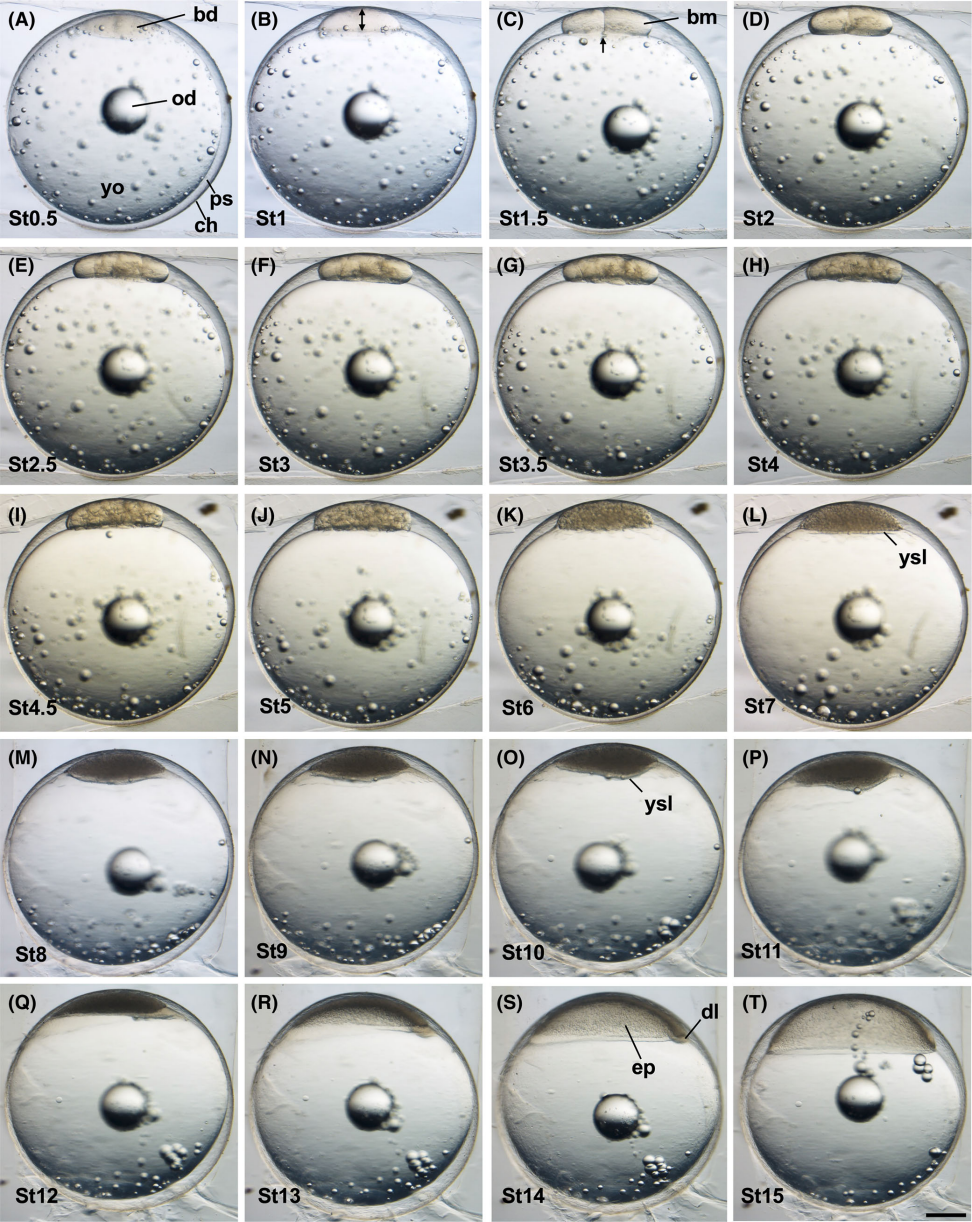
Normal embryonic development in *Aphanius dispar* from cleavage to early gastrula stages. Lateral view showing stage transition from cleavage (A–I) to blastula (J–L) to gastrula (Q–T). Stage time (hpf) is shown at the bottom left of each picture. bd, blastodisc; bm, blastomere; ch, chorion; dl, dorsal lip; ep, epiboly; od, oil droplet; ps, perivitelline space; yo, yolk; ysl, yolk syncytial layer Scale bar = 500 μm. Embryos were raised at 28°C.

**FIGURE 2:**
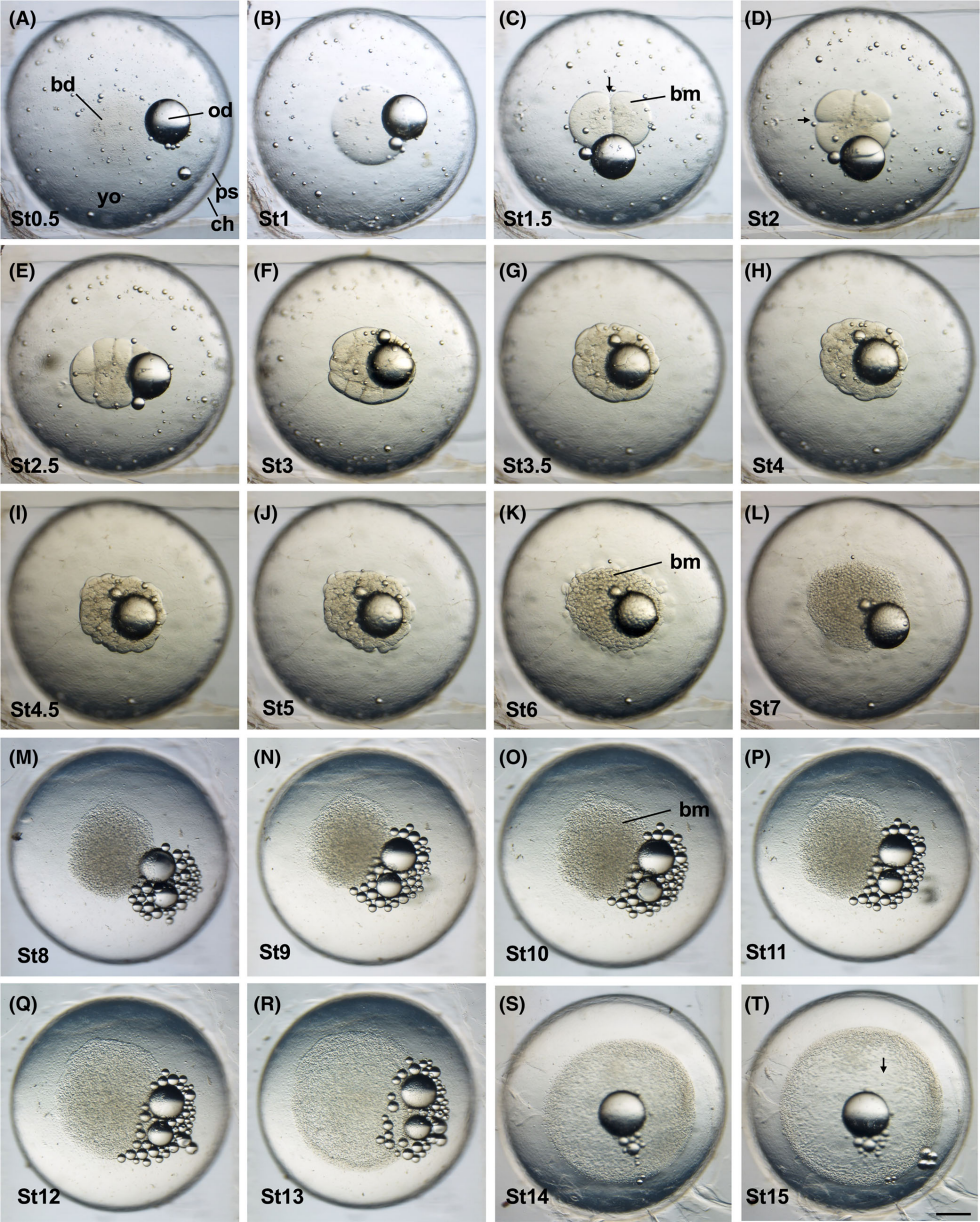
Normal embryonic development in *Aphanius dispar* from cleavage to early gastrula stages. Animal pole view showing stage transition from cleavage (A–I) to early to mid‐blastula (J–L) to late blastula (M‐P) to gastrula (Q–T). Stage time (hpf) is shown at the bottom left of each picture. bd, blastodisc; bm, blastomere; ch, chorion; dl, dorsal lip; ep, epiboly.; od, oil droplet; ps, perivitelline space; yo, yolk. Scale bar = 500 μm. Embryos were raised at 28°C.

A thin and single blastodisc is visible at the animal pole (Figures 1A and 2A). The chorion is elevated and separated from the egg. However, the size of the chorion is only slightly larger than the one of the eggs itself. Therefore, the perivitelline space is relatively small. There is normally a single large oil droplet observed with many other smaller droplets in the yolk.

#### 2.1.2 Stage 1 (1 hpf): late 1‐cell stage

The blastodisc becomes swollen and bigger (Figures 1B and 2B). The size of the blastodisc is indicated by a double‐headed arrow in the lateral view of the embryo (Figure 1B).

#### 2.1.3 Stage 1.5 (1.5 hpf): 2‐cell stage

The first cleavage produces two rounded blastomeres of equal size (Figures 1C and 2C). The cleavage is indicated by an arrow.

#### 2.1.4 Stage 2 (2 hpf): 4‐cell stage

Cleavage occurs in the two‐cell blastomere at a right angle to the first cleavage (Figures 1D and 2D). It produces four rounded blastomeres of the same size in a 2 × 2 array.

#### 2.1.5 Stage 2.5 (2.5 hpf): 8‐cell stage

In this stage, cleavages occur in two separate planes; they are parallel to the first one to cut the blastodisc into a 2 × 4 array of blastomeres, forming eight blastomeres (Figures 1E and 2E).

#### 2.1.6 Stage 3 (3 hpf): 16‐cell stage

The fourth cleavage occurs along two planes to produce a 4 × 4 array of cells (Figures 1F and 2F). All the cells are arranged in two tiers on top of the yolk.

#### 2.1.7 Stage 3.5 (3.5 hpf): 32‐cell stage

The 32 blastomeres are present in arrays, with more cells present in the upper tier (Figures 1G and 2G).

#### 2.1.8 Stage 4 (4 hpf): 64‐cell stage

In this stage, the sixth cleavage plane, the blastomeres are dividing and decreasing in size, producing a mass with less rounded edges (Figures 1H and 2H).

#### 2.1.9 Stage 4.5 (4.5 hpf): 128 cell stage

The seventh cleavage further reduces the size of the dividing blastomere (Figures 1I and 2I).

#### 2.1.10 Stages 5, 6, and 7 (5, 6, and 7 hpf): early to mid‐blastula stages

The eighth, ninth, and tenth cleavages. At these stages, the marginal cells fuse to the yolk and start to form the yolk syncytial layer (ysl) (Figures 1J–L and 2J–L).

#### 2.1.11 Stages 8, 9, 10, and 11 (8, 9, 10, and 11 hpf): late blastula stages

The cells of the blastomere are smaller than in the mid‐blastula stage, and the blastodisc is clearly flattened (Figures 1M–P and 2M–P). The marginal ysl becomes clearly visible.

#### 2.1.12 Stages 12 and 13 (12 and 13 hpf): late blastula stages

The blastoderm starts to expand (Figures 1Q, R and 2Q, R).

#### 2.1.13 Stage 14 (14 hpf): early gastrula stage

Dorsal marginal area becomes thicker to form the embryonic shield/dorsal lip (Figures 1S and 2S).

#### 2.1.14 Stage 15 (15 hpf): early gastrula stage

The blastoderm further expands (Figures 1T and 2T). The cell density in the animal pole area reduces, indicated by an arrow (Figure 2T).

#### 2.1.15 Stage 16 (16 hpf): 25% epiboly stage

**FIGURE 3:**
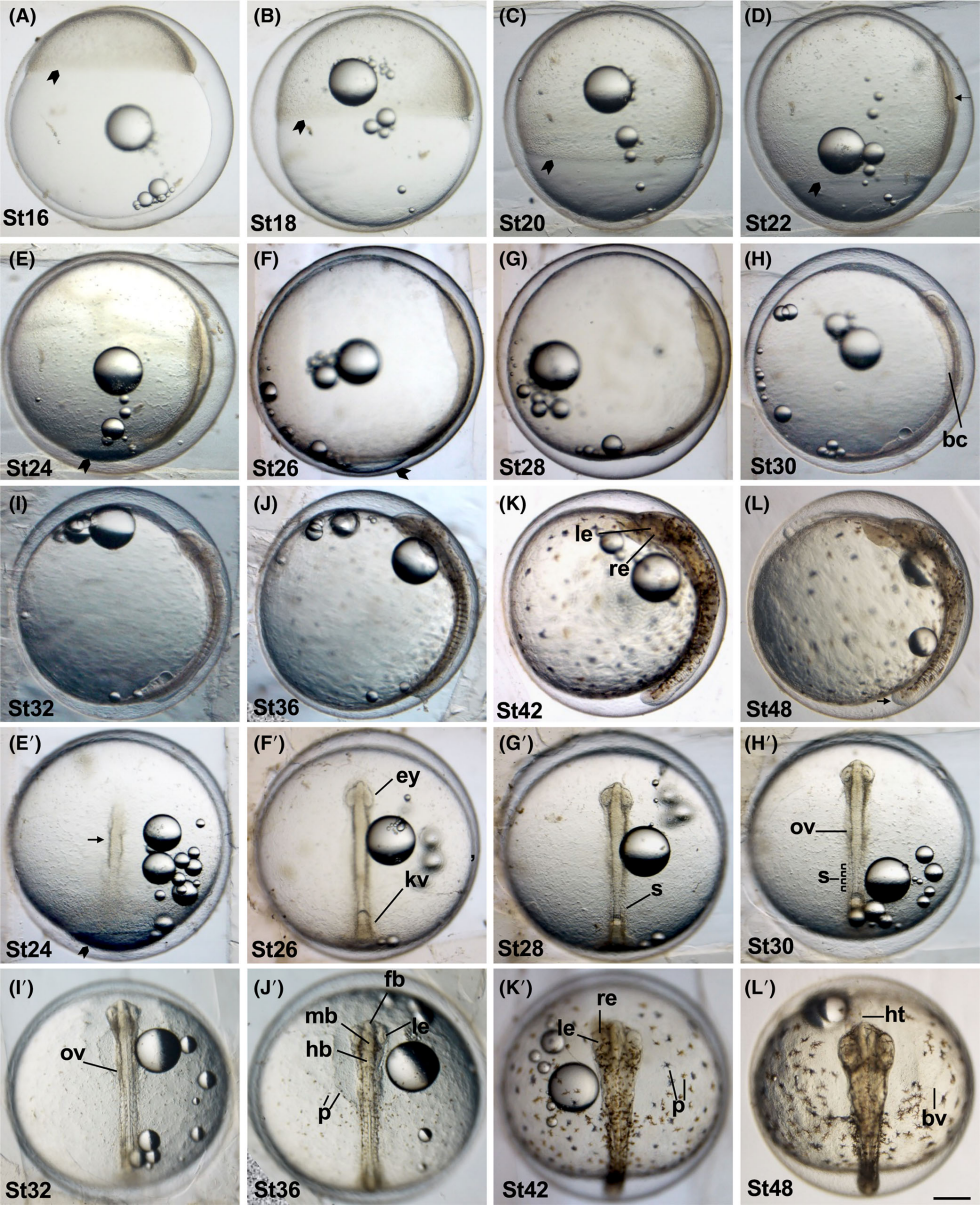
Developmental stages in *Aphanius dispar* show stages of gastrulation, somitogenesis, and organogenesis. (A–L) lateral views of developed embryos. (E′–L′) Views of the head stage time (hpf) are shown at the bottom left of each picture. Arrowhead indicates the percentage of epiboly development at (A) 25%, (B) 50%, (C) 75%, (D) 90% and (E) 100%. bc, body cavity; bv, blood vessels ep, epiboly; ey, eye; fb, forebrain; hb, hindbrain; ht, heart tube; l, lens; kv, Kupffer's vessicle; mb, midbrain; ov, otic vesicle; p, pigmentation; re, retina; s, somite. Scale bar = 500 μm. Embryos were raised at 28°C.

Epiboly gradually advances toward the vegetal pole and covers 25% of the yolk sphere (Figure 3A). The percentage of epiboly is indicated by an arrowhead.

#### 2.1.16 Stage 18 (18 hpf): 50% epiboly stage

Epiboly progressively advances and covers half of the yolk sphere (Figure 3B). The percentage of epiboly is indicated by an arrowhead.

#### 2.1.17 Stage 20 (20 hpf): 70% epiboly stage

The blastoderm covers about two‐thirds of the yolk sphere (Figure 3C). The percentage of epiboly is indicated by an arrowhead.

#### 2.1.18 Stage 22 (22 hpf): 80% epiboly stage

The thin line of the dorsal body axis (indicated by the arrow) starts to become visible (Figure 3D). The percentage of epiboly is indicated by an arrowhead.

#### 2.1.19 Stage 24 (24 hpf): 90% epiboly stage

The blastoderm covers around 90% of the yolk sphere (Figure 3E, E′). The dorsal body axis becomes longitudinal and is clearly defined as a narrow streak (indicated by an arrow). The percentage of epiboly is indicated by an arrowhead.

#### 2.1.20 Stage 26 (26 hpf): 95% epiboly stage

The blastoderm almost covers the yolk (Figure 3F, F′). A small germ ring surrounding the open yolk is still visible near the vegetal pole. The dorsal body axis is clearly visible. Eye fields are split in left and right. Kupffer's vesicle is also formed and visible. The percentage of epiboly is indicated by an arrowhead.

#### 2.1.21 Stage 28 (28 hpf): 100% epiboly stage

The blastoderm covers the entire yolk. There is a long bulge on the dorsal surface of the egg due to the presence of the embryo. The first two somites are visible at this stage.

#### 2.1.22 Stage 30 (30 hpf): 3/4‐somite stage

The embryonic body increases in size longitudinally and laterally to become more distinct (Figure 3H, H′). Three to four pairs of somites are visible. The outline of the body cavity (bc) on the yolk at both sides of the hindbrain becomes visible.

#### 2.1.23 Stage 32 (32 hpf): 6‐somite stage

At this stage, the otic vesicle can clearly be observed (Figure 3I, I′). The bc is expanded and becomes clearer. Faint signs of pigmentation start appearing in the dorsal half of the yolk.

#### 2.1.24 Stage 36 (36 hpf )

Three brain regions (forebrain, midbrain, and hindbrain) are recognizable (Figure 3J, J′). Pigmentations with two pigment cells (brown fluoroleucophores and black melanophores) are clearly distinguished and widely distributed in the embryo and yolk. The lenses are clearly identifiable. Somites in the trunk region are also clearly distinguishable.

#### 2.1.25 Stage 42 (42 hpf )

Midbrain (tectum) and hindbrain are enlarged, and the furrow is noticeable in the mid‐hindbrain boundary (Figure 3K, K′). The lens and retina are clearly distinguishable in the eye. Pigment cells (melanophores and leucophores) start to form dendritic shapes. The tubular heart is visible underneath the head. The heart is starting to beat at this stage.

#### 2.1.26 Stage 48 (48 hpf )

The blood vessels are formed in the yolk, and blood circulation is apparent (Figure 3L, L′). The tubular heart becomes clearly visible. The three brain sections are increased in size and have become more apparent. The ventral side of the tail is still attached to the yolk (indicated by the arrow).

#### 2.1.27 Stage 60 (60 hpf )

**FIGURE 4:**
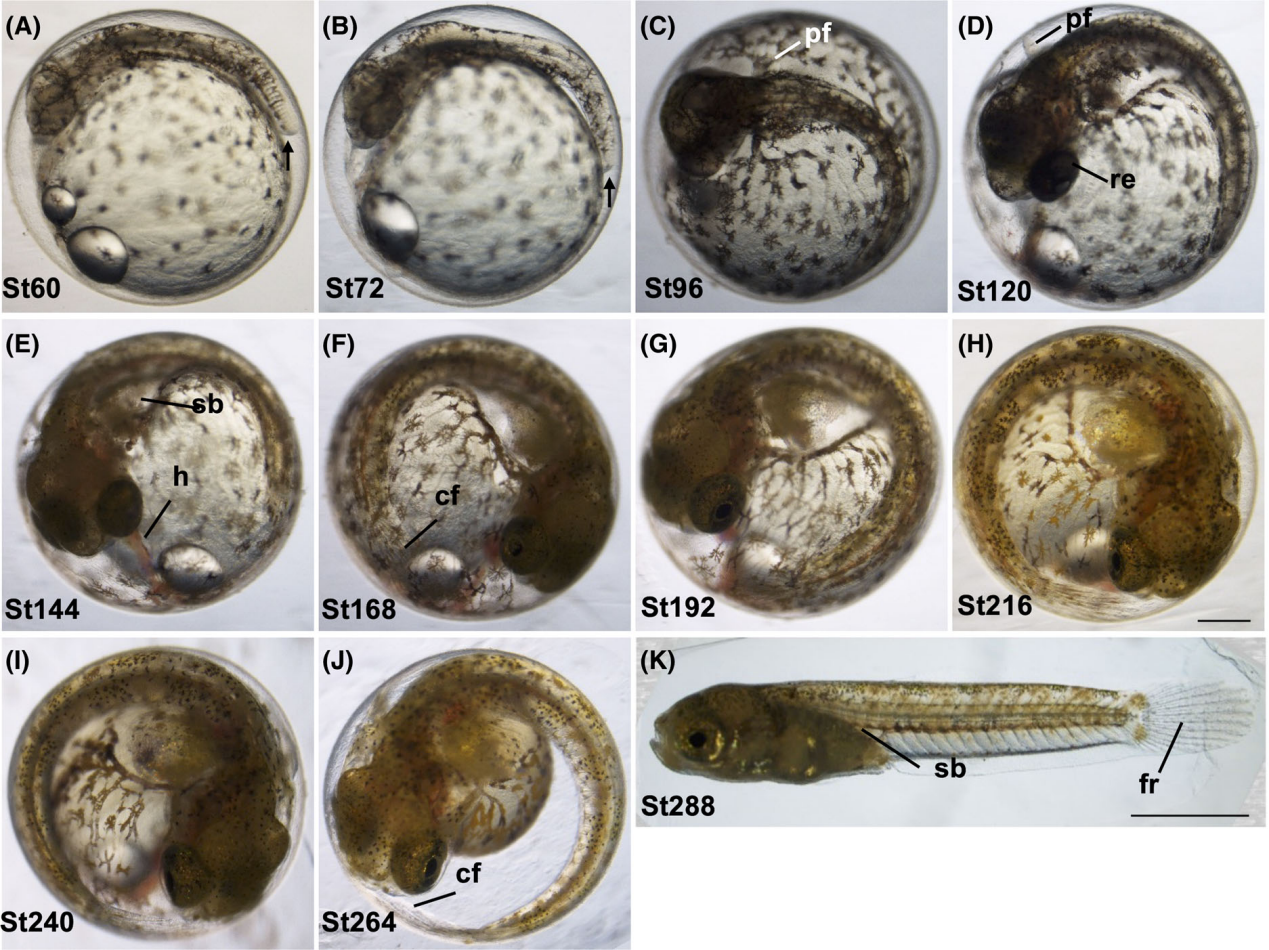
Development stages from organogenesis to hatching. (A–J) Lateral views of developed embryos at late stages. Panel (K) shows the dechorionated embryo. Stage time (hpf) is shown at the bottom left of each picture. cf, caudal fin; fr, fin rays; h, heart; pf, pectoral fin; re, retina; sb, swim bladder. Scale bar = 500 μm (A–J), 2 mm (K). Embryos were raised at 28°C.

**FIGURE 5:**
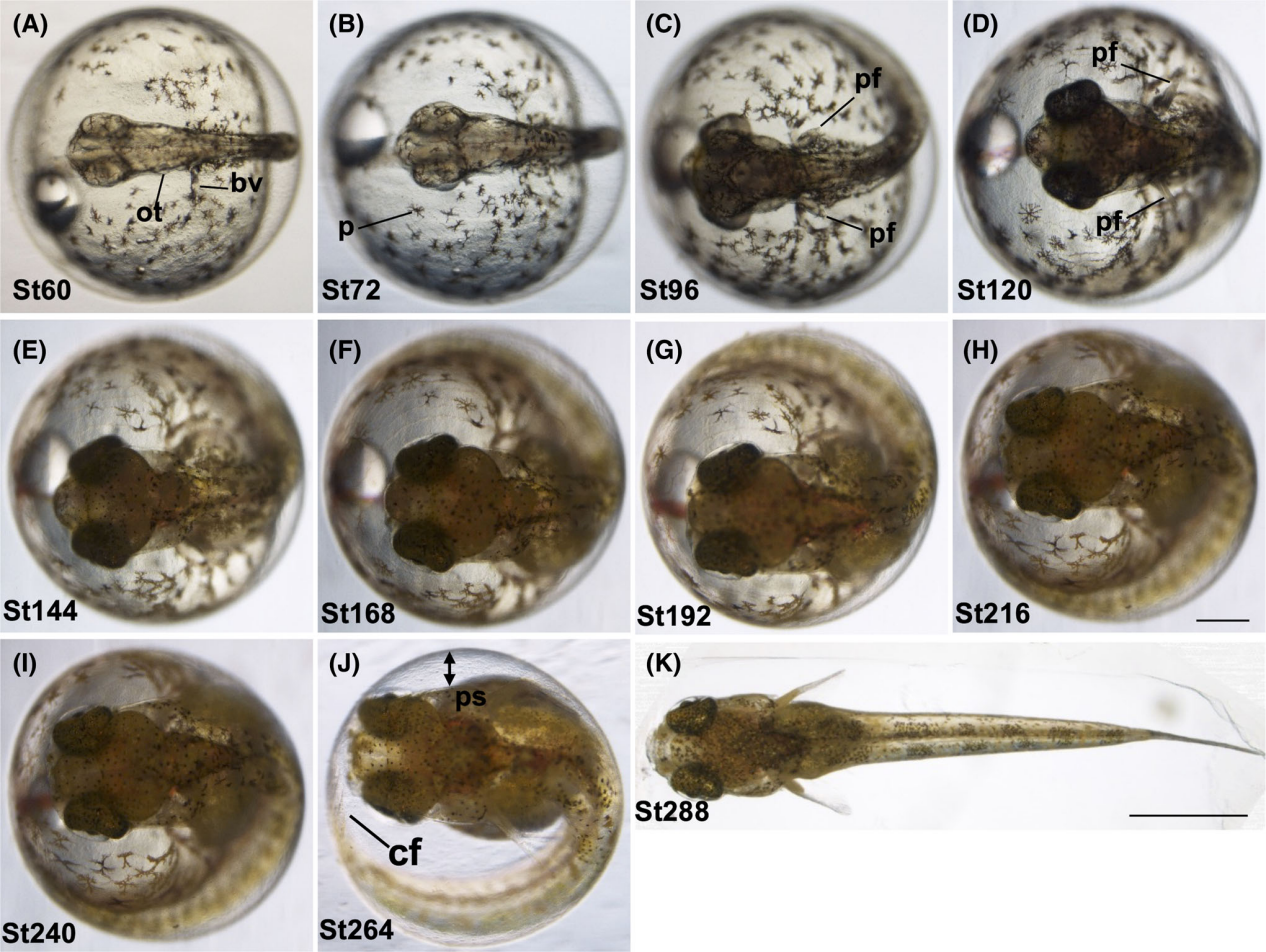
Development stages from organogenesis to hatching. (A–J) Dorsal views of developed embryos at late stages. (K) is dechorionated embryos. Stage time (hpf) is shown at the bottom left of each picture. bv, blood vessels; cf, caudal fin; ot, otolith; p, pigmentation; pf; pectoral fin; ps, perivitelline space. scale bar = 500 μm (A–J), 2 mm (K). Embryos were raised at 28°C.

The embryonic body enlarges; otoliths appear within the otic vesicle (Figures 4A and 5A). The blood vessels are increased in size, and circulation is visible in two blood vessels within the embryonic body. The anlage of the liver appears. The tail is elongated, and the tip of the tail is no longer attached to the yolk (indicated by an arrow). The mobility of the body increases, pigment cells grow, and the pectoral fin starts to form.

#### 2.1.28 Stage 72 (72 hpf )

The head becomes larger and the tail is further elongated (Figures 4B and 5B). Body movement is apparent, especially in the tail. Pigmentation in the eyes is still not developed. Pigment cells in the yolk are increased in size.^23^

#### 2.1.29 Stage 96 (96 hpf )

Eyes are partly pigmented (Figures 4C and 5C). The pectoral fins come throughout the middle of the lateral surface of the trunk. Liver bud starts to form.

#### 2.1.30 Stage 120 (120 hpf): enlarged embryonic body

The body and head increase in size (Figures 4D and 5D). The eyes begin to enlarge, and the retina of the eyes begins to become dark due to melanization. Pectoral fins are more clearly visible. The tail fin fold is developed to form a round shape, but fin rays are not visible in it.

#### 2.1.31 Stage 144 (144 hpf )

From this stage, two distinctively larger brown pigment cells (fluoroleucophore^23^) appear at the border between the tail and caudal fin (Figures 4E and 5E). The caudal fin forms, with fin rays starting to become visible. The swim bladder starts to form. Blood circulation in the heart is clearly visible.

#### 2.1.32 Stage168 (168 hpf )

The blood circulates in the caudal and dorsal fins (Figures 4F and 5F). The air bladder becomes clearer, and pigment cells appear in the caudal fin. Early dorsal fin/dorsal fin fold is now visible. The fringe of the caudal fin turns into a wavy pattern.

#### 2.1.33 Stages 192 and 216 (192 and 216 hpf )

Movement of the eyes is clearly seen (Figures 4G, H and 5G, H). Around this stage, the yolk mass starts to show a clear decrease.

#### 2.1.34 Stages 240 and 264 (240 and 264 hpf )

The yolk mass starts to noticeably decrease with increasing perivitelline space (Figures 4I, J and 5I, J). Jaw forms but with no movement. Enlargement of the eyes occurs with constant movement, and the caudal fin reaches the head. Increases occur in the mobility of the fins and the whole body.

#### 2.1.35 Stage 288 (288 hpf): hatching

The whole body is developed, and the swim bladder expands remarkably (Figures 4K and 5K). Fin rays are observed clearly, along with the typical patterns of pigmentation of the adult fish. The mouth moves easily, allowing the fish to tear the chorion and escape through the body and tail movements.

### 2.2 Measurement of growth rate of *A. dispar*

**FIGURE 6:**
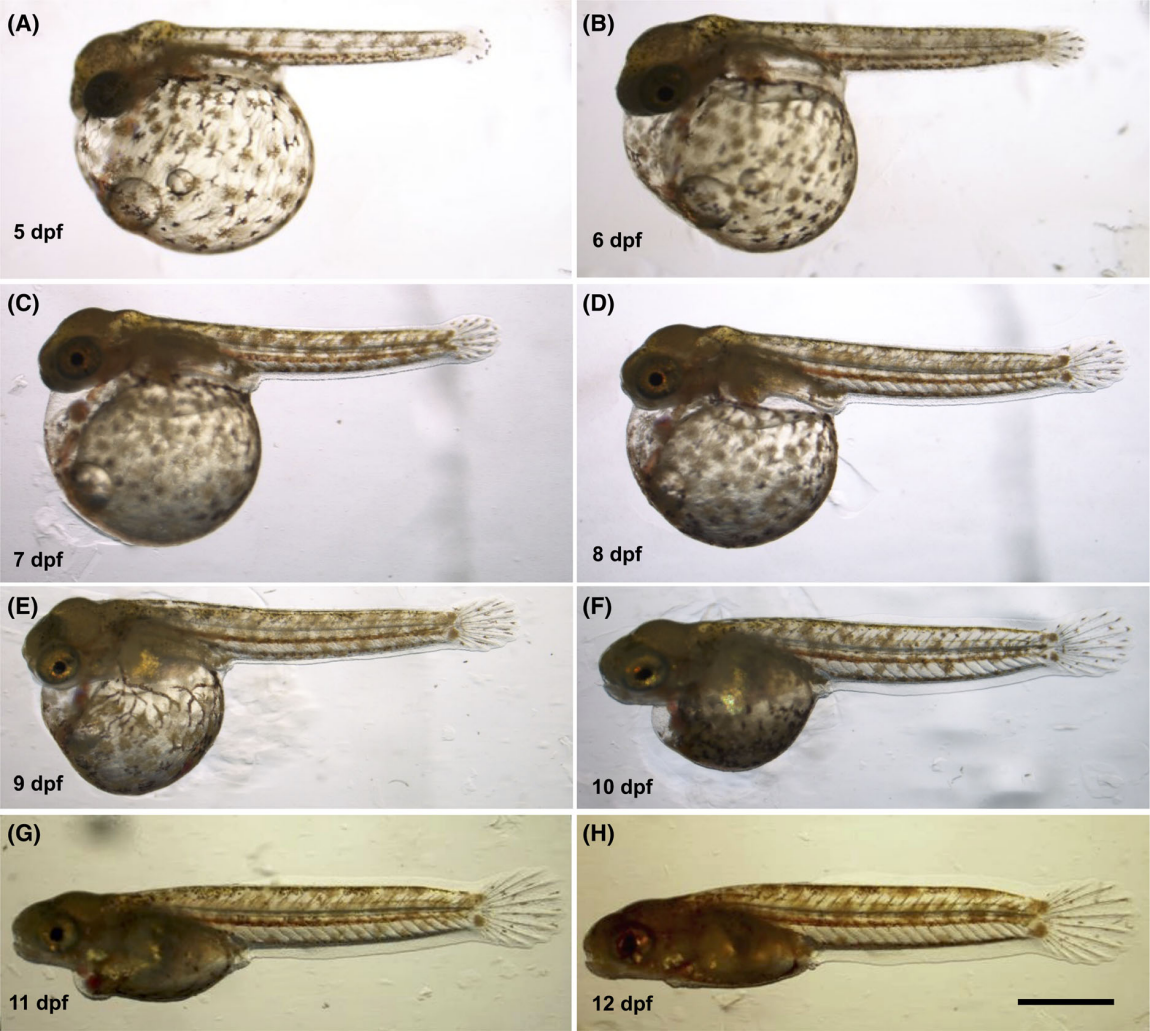
Lateral view of the development of dechorionated *Aphanius dispar* embryos. Lateral view (A–H). Embryos were enzymatically dechorionated and imaged from a lateral view from 5 dpf to the hatching day. The scale bar = 2 mm. Embryos were raised at 28°C.

**FIGURE 7:**
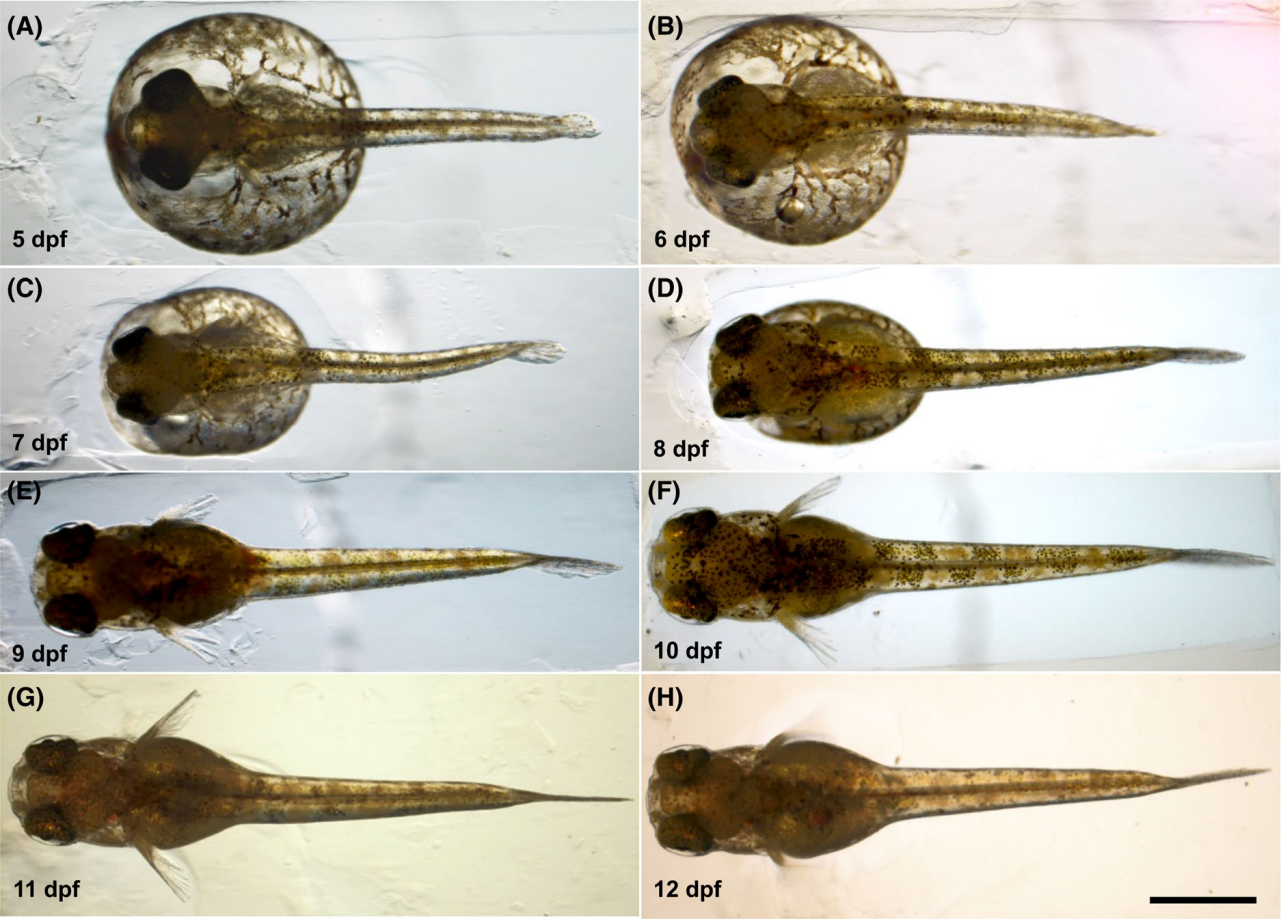
Dorsal view of the dechorionated *Aphanius dispar* embryos. Dorsal view (A‐H). Embryos were enzymatically dechorionated and imaged from dorsal view from 5 dpf to the hatching day. Scale bar = 2 mm. Embryos were raised at 28°C.

**FIGURE 8:**
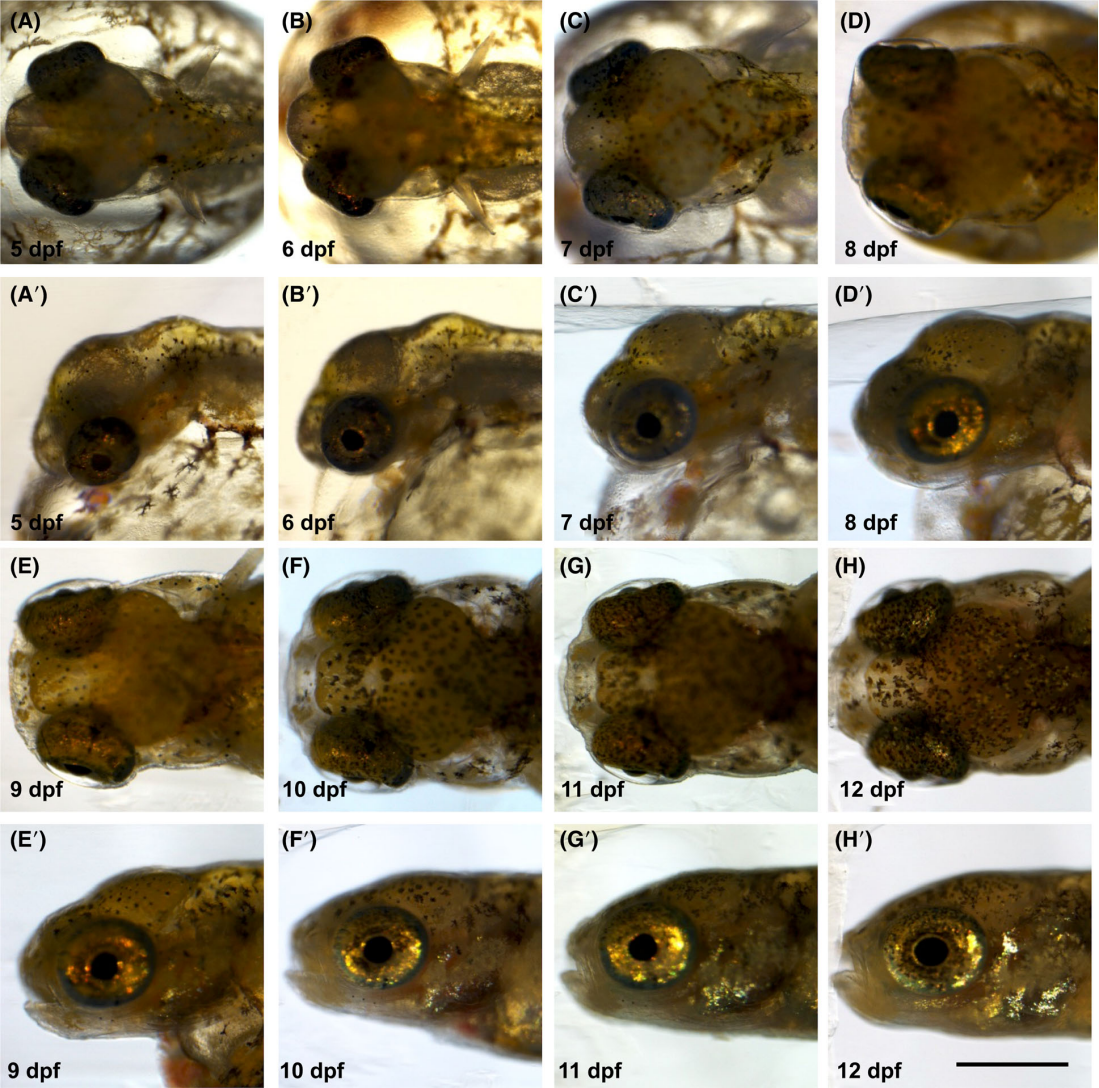
Head of dechorionated *Aphanius dispar* embryos from Day 5 to the hatching day. Lateral view (A–H) and dorsal view (A′–H′) of developed head embryos from Day 5 to 12 days. Scale bar = 200 μm. Embryos were raised at 28°C.

**FIGURE 9:**
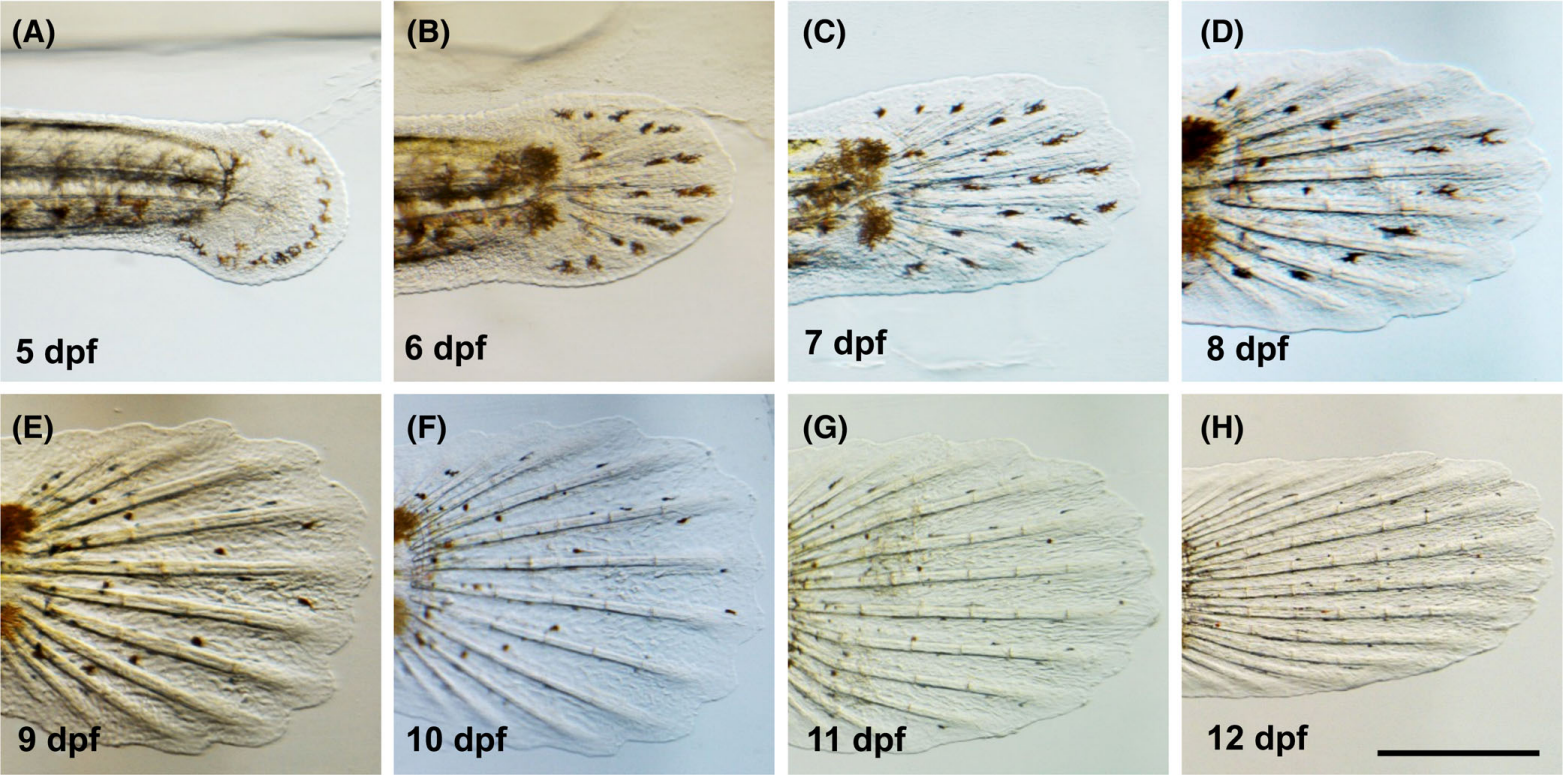
The tail fin length of dechorionated *Aphanius dispar* embryos. Lateral views (A–H) of developed tail‐length embryos from Day 5 to Day 12, the hatching day. Scale bar = 200 μm. Embryos were raised at 28°C.

**FIGURE 10:**
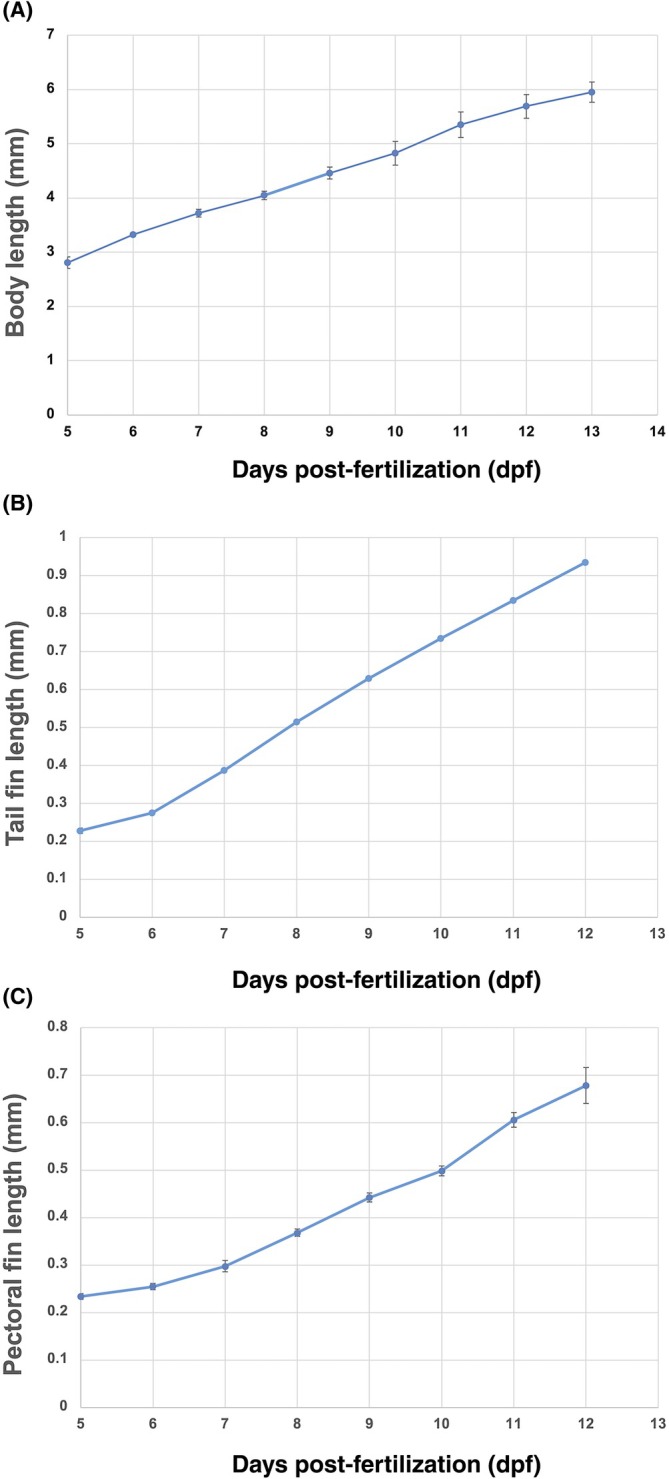
Measurements of length of body, tail fin, and pectoral fin of *Aphanius dispar* embryos. Graphs show the gradual increase in the (A) length of body, (B) tail fin, and (C) pectoral fin from 5 to 12 dpf. The *x*‐axis represents length in mm, while the *y*‐axis shows days post‐fertilization (dpf). Embryos were raised at 28°C.

To better visualize and quantitatively analyze the growth of the embryos, the chorion was enzymatically removed (pronase and hatching enzyme [HE]), allowing measurements of the length of the body, tail fin, and pectoral fin from 5 to 12 dpf (Table 1) (Figures 6, 7, 8, 9, 10). During this period, the length increased from 2.8 to 6.0 mm. Tail and pectoral fin length were increased from 230 to 1030 and 240 to 730 μm, respectively. The daily rate of increase in these metrics is highly linear and constant. The growth rates of the length of the body, tail and pectoral fins were 400, 100, and 60 μm/day, respectively.

**TABLE 1:** Body, pectoral fin, and tail fin growth in *Aphanius dispar* larvae between 5 and 12 dpf.

		Length measurements (mm)		
		Body^A^	Pectoral fin^B^	Tail fin^C^
dpf	5	2.808	0.234	0.228
	6	3.323	0.255	0.275
	7	3.721	0.298	0.386
	8	4.048	0.368	0.515
	9	4.460	0.442	0.629
	10	4.823	0.498	0.734
	11	5.350	0.606	0.835
	12	5.688	0.678	0.934

*Note*: The table shows the significant difference in measurement of (A) body length (*p* < .001, *F* = 47.9); (B) pectoral fin (*p* < .001, *F* = 102.8); and (C) tail fin at different days (*p* < .001, *F* = 58.8).

### 2.3 Influence of water temperature on embryonic development of *A. dispar*

**FIGURE 11:**
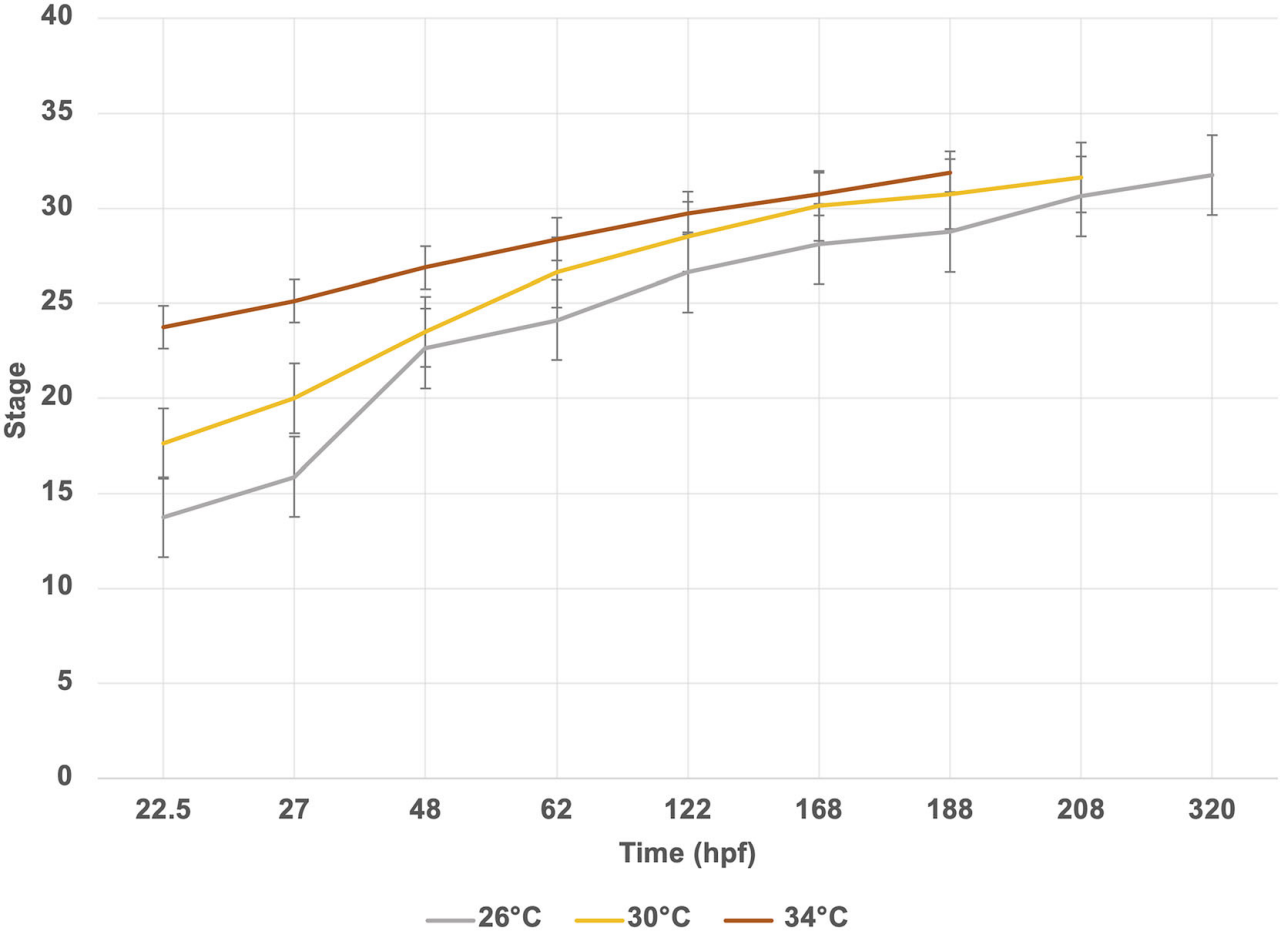
Influence of water temperature on the growth of *Aphanius dispar* embryos. Embryonic development time was investigated at a range of temperatures. The normal embryonic developmental time was recorded from fertilization. Each data point represents the mean of three biological replicates ±SE. *p*‐Values between .05 and .001 were considered significant.

**FIGURE 12:**
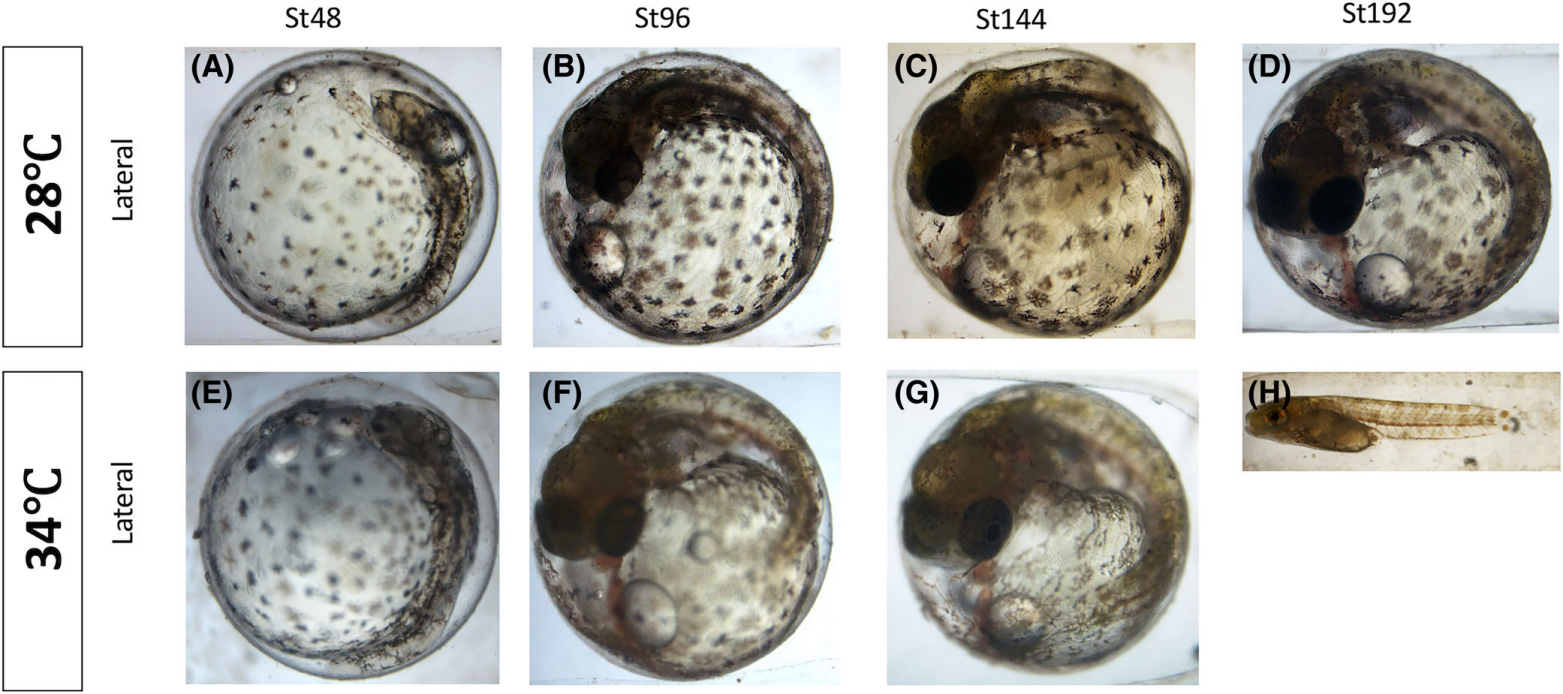
Representative images of embryos incubated at 28 and 34°C at St48, St96, St144, and St192. Lateral views (A–H). Hours post‐fertilization is indicated on the top side of the embryos.

**FIGURE 13:**
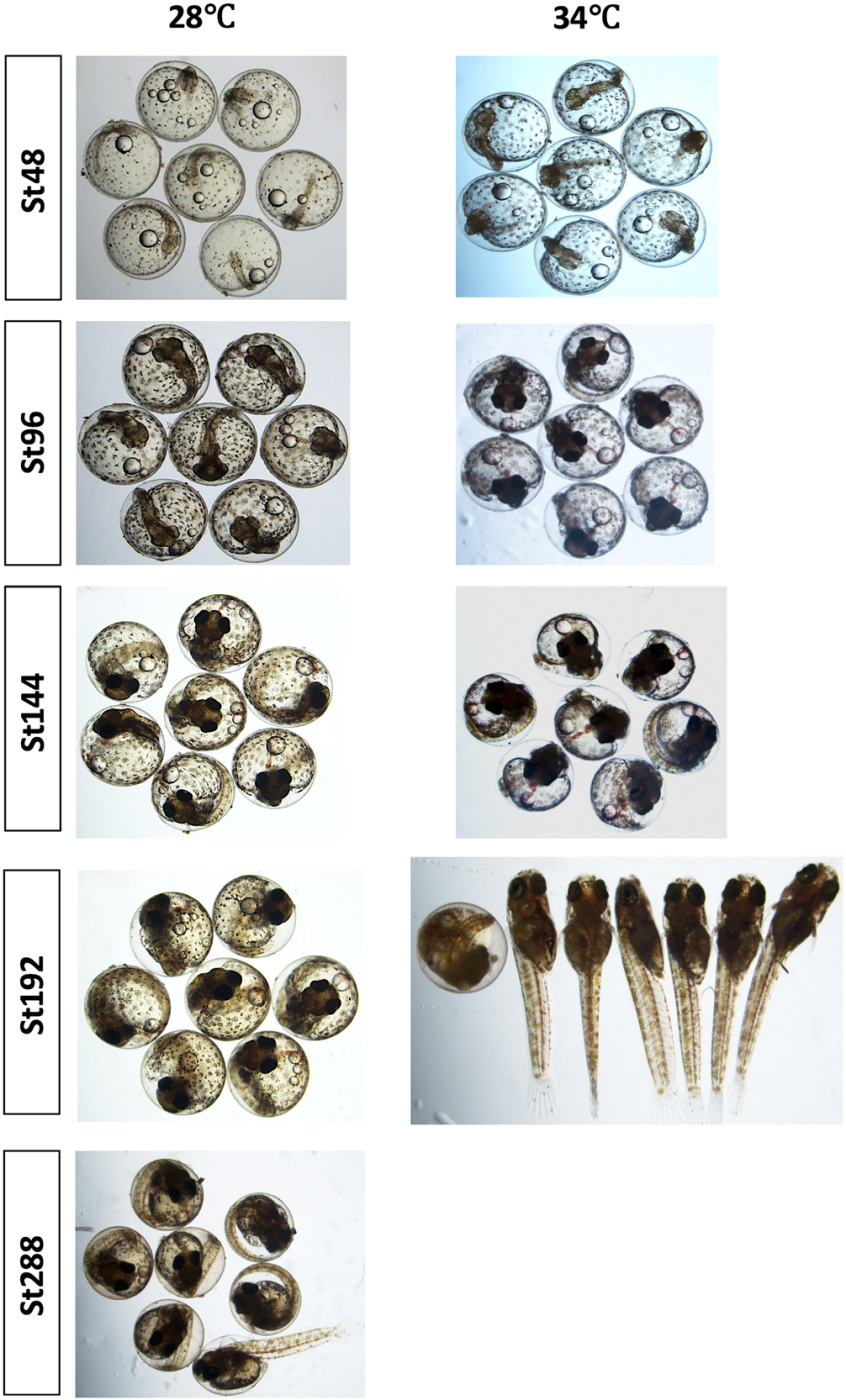
Group images of embryos incubated at normal (28°C), and higher temperature (34°C) at St48, St96, St144, St192, and St288. Hours post‐fertilization are indicated on the left‐hand side. Embryos incubated at higher 34°C hatched earlier around St192, while embryos incubated at 28°C hatched at 288 hpf.

To examine how *A. dispar* adapted to different thermal environments, normal embryonic development was studied across a range of temperatures (26–34°C). Staging curves of embryonic development were generated from fertilization to the hatching stage (Figure 11). *A. dispar* fertilized eggs were incubated at 26, 30, and 34°C. At 30 and 34°C, hatching fell between these temperatures, occurring at 208 and 188 hpf, respectively, compared to the typical 288 hpf for control conditions (28°C) (Figures 11, 12, 13).

## 3 DISCUSSION AND CONCLUSION

In this study, the embryonic development processes in *A. dispar* were examined under “normal” environmental conditions at 28°C. The development stages were defined according to the hours post‐fertilization at 28°C water temperature. We have demonstrated that *A. dispar* embryo is optically transparent, making it a useful model for monitoring normal and abnormal embryonic development. The overall embryonic development of *A. dispar* is very similar to that seen in other killifish species such as mummichog,^24^ mangrove killifish,^25^, ^26^ and turquoise killifish^27^ and with other species such as medaka rice fish.^28^ However, unlike turquoise killifish and medaka, the chorion of *A. dispar* embryo does not have hairs and therefore optical accessibility to the embryo is ideal. At the early stages of *A. dispar* development, many small oil droplets are also observed in the transparent yolk, which becomes less apparent at the later stages. While in the mummichog and mangrove killifish, a considerable number of oil droplets are noticeable at the gastrula, somitogenesis, and organogenesis stages of the fish embryo that disturb capturing high resolution images of the embryos.^29^, ^30^ Considering the smooth and transparent chorion without hairs and the small number of oil droplets, *A. dispar* is more suitable for imaging analyses compared with many other related species. In addition, medaka embryos show rhythmic contraction during which the whole embryo body shows contractive movement every minute^31^ making time‐lapse imaging of these embryos challenging. In the case of the *A. dispar* embryo, such movement was not observed; therefore, long time‐lapse imaging is possible.

Though the overall morphology of developing embryos is like other killifish and rice fish species, the pigmentation pattern shows large variability. In medaka, mangrove killifish and mummichog, pigment cells only appear after the completion of somitogenesis, but in *A. dispar*, black pigment (melanophore) and brown pigment (fluoroleucophore) appear at the onset of somitogenesis.^23^ Such early appearance of the pigment cells may be due to the adaptation of the species to living in an environment of strong sunlight often with limited vegetation cover in the Middle East.

The data presented reveal that the embryonic developmental time was affected by high temperatures and that this could result in a reduction of 2–3 days in hatching time due to accelerated development rates.

Moreover, we previously demonstrated that CRISPR/Cas9 has very high efficiency in editing the genomes of *A. dispar* embryos. This makes it an excellent model for studying genetic disease modeling using genome editing techniques.^23^ Therefore, besides their value as a toxicological study model, *A. dispar* embryos might serve as an alternative model for in vivo studies for infection, cancer, immunological responses genetic diseases, and drug testing for a longer period of time.

## 4 EXPERIMENTAL PROCEDURE

### 4.1 *A. dispar* care and maintenance

All animal procedures were approved by the Biosciences Ethics Committee at the University of Exeter's Faculty of Health and Life Sciences. Wild‐type (WT) *A. dispar*, was maintained and bred in a recirculating system in the Aquatic Resource Centre. *A. dispar* eggs were collected by natural spawning within 1 h of morning light initiation (14:10 light:dark cycle) in the breeding chambers and transferred (30 per dish) to 10 cm Petri dishes containing artificial salt water (ASW) with 35 ppt salinity, typically incubated at 28°C. The eggs were then subjected to incubation to complete their embryonic development. For specific experiments, embryos were collected from the breeding tanks and incubated at temperatures ranging from 26 to 34°C (26, 28, 30, and 34°C), with the incubation starting within 1 h of collection.

### 4.2 Dechorionation

Dechorionation was conducted with some modification of a method previously used in other species.^29^, ^32^ To prepare HE extract, 20 *A. dispar* embryos at stage 96 (just before hatching) were homogenized in 200 μL ASW and placed overnight at 4°C in Eppendorf tubes with slow rotation. The supernatant was collected after centrifugation at 14,000 rpm for 10 min. The HE extracts were diluted two‐fold with ASW and stored at −20°C. For dechorionation, eggs were cleaned and transferred on fine sandpaper (p2000 grit size), then gently rolled with a finger for 1 min to scar the chorion surface. Embryos were then incubated for 1 h with 10 mg/mL pronase (Sigma) at 28°C. Subsequently, embryos were washed in ASW to remove traces of pronase. HE was thawed and centrifuged at 14,000 rpm for 10 min, and the supernatant was added to the embryo for 2–6 h at 28°C. After dechorionation, embryos were transferred gently to another dish of fresh ASW (35 ppt). Used HE was frozen at −20°C and re‐used for dechorionation a few times.

### 4.3 Imaging and analyses

*A. dispar* embryo and larvae images were acquired using a Nikon DS‐L3 (DS‐Fi2‐L3u) camera and NIS‐Elements software (version 4.30) on a Nikon SMZ1500 microscope. Embryo development was monitored daily throughout the experimental period, from fertilization until 12 days post‐fertilization (12 dpf). Embryos with chorion were placed in a custom made agarose mold with artificial sea water, oriented and imaged. The dechorionated embryos were anesthetized with MS222 (0.4%) in ASW and mounted in 2% methylcellulose. Body, tail fin, and pectoral fin measurements were made on three embryos, as well as the effects of temperature on embryo size, were subsequently quantified using ImageJ software. For comparison of survival rates, we used three biological replicates, each containing seven embryos. Embryos were terminated at the time when embryos came out of the chorion. Standard error of mean was calculated for three biological replicates.

## FUNDING INFORMATION

Amena Alsakran was funded by a PhD studentship from the Prince Sattam Bin Abdulaziz University Kingdom of Saudi Arabia. Atyaf Hamied was funded by a PhD studentship from the University of Baghdad, Iraq Government. Rashid Minhas, Mark Ramsdale, and Tetsuhiro Kudoh are funded by NC3R grant number NC/X001121/1. We acknowledge funding from the MRC Centre for Medical Mycology at the University of Exeter (MR/N006364/2 and MR/V033417/1), the NIHR Exeter Biomedical Research Centre, MRC Doctoral Training Grant MR/W502649/1, and the Biotechnology and Biological Sciences Research Council (BB/D005108/1) to Rod W. Wilson. For the purpose of open access, the author has applied a Creative Commons Attribution (CC BY) licence to any Author Accepted Manuscript version arising from this submission.

## CONFLICT OF INTEREST STATEMENT

The authors declare that they have no competing interests.
